# Silicone Composites with CNT/Graphene Hybrid Fillers: A Review

**DOI:** 10.3390/ma14092418

**Published:** 2021-05-06

**Authors:** Marie N. Barshutina, Valentyn S. Volkov, Aleksey V. Arsenin, Albert G. Nasibulin, Sergey N. Barshutin, Alexey G. Tkachev

**Affiliations:** 1 Center for Photonics and 2D Materials, Moscow Institute of Physics and Technology, 141700 Dolgoprudny, Russia; arsenin.av@mipt.ru; 2 NanoTechCenter Ltd., 392000 Tambov, Russia; postmaster@kma.tstu.ru; 3 Skolkovo Institute of Science and Technology, Nobel Str. 3, 121205 Moscow, Russia; a.nasibulin@skoltech.ru; 4 School of Chemical Engineering, Aalto University, P.O. Box 16100, 00076 Aalto, Finland; 5 Department of Technology and Methods of Nanoproducts Manufacturing, Tambov State Technical University, 392000 Tambov, Russia; barshutin.sn@tstu.ru

**Keywords:** carbon nanotubes, graphene, hybrid materials, silicone composites

## Abstract

This review is dedicated to versatile silicone rubber composites based on carbon nanotube/graphene (CNT/G) hybrid fillers. Due to their unique mechanical, electrical, thermal, and biological properties, such composites have enormous potential for medical, environmental, and electronics applications. In the scope of this paper, we have explored CNT/graphene/silicone composites with a different morphology, analyzed the synergistic effect of hybrid fillers on various properties of silicone composites, and observed the existing approaches for the fabrication of hybrid composites with a seamless, assembled, and/or foamed structure. In conclusion, current challenges and future prospects for silicone composites based on CNTs and graphene have been thoroughly discussed.

## 1. Introduction

In the last two decades, carbon nanotubes/graphene (CNT/G) hybrid structures have been investigated as promising fillers for manufacturing of highly homogeneous composites with potential applications in diverse fields of research including electronics, supercapacitors, batteries, solar cells, sensors, and biosensors [1,2]. The idea of creating CNT/G hybrid structures was first introduced by Matsumoto and Saito in 2002 [3]. Since then, the number of research works in this area has been steadily growing due to the unique properties of CNT/G hybrid materials. In particular, many studies demonstrated the synergistic effect of CNT/G fillers on the mechanical, electrical, thermal, and electromagnetic properties of polymer composites [4,5,6,7]. It was reported that the synergy between graphene and CNTs facilitates their dispersion in polymer composites, maximizes the contact surface area of filler particles with polymer matrices, and promotes strong filler-matrix interfacial interactions. As a result, hybrid polymer composites based on graphene and CNTs exhibit superior properties compared to composites with either of these fillers alone.

To date, various types of polymers have been used to prepare CNT/G hybrid composites [8,9,10], and among them, silicone rubber is believed to be one of the most advantageous [11,12]. Silicone rubber (or polydimethylsiloxane, PDMS) is well-known for its high flexibility, chemical inertness, optical transparency, heat resistance, and good biocompatibility. The reinforcement of PDMS with CNT/G hybrid fillers enables to obtain highly conductive and stretchable materials, which could be used for multiple applications in medicine, environment protection, and wearable electronics. A number of studies were reported so far on the fabrication of CNT/G hybrid silicone composites comprising different types of CNTs (single- (SWCNTs), few-, or multi-walled (MWCNTs)) and graphene (graphene oxide (GO), 2D planar graphene, 3D graphene foam (GF) [13], graphene nanoscrolls [14]). All of these studies were thoroughly reviewed in this paper in order to summarize and introduce the following aspects: (a) the nanoscale architecture of CNT/G hybrid fillers; (b) strategies for the preparation of CNT/G/PDMS composites; (c) the synergistic effect of CNT/G hybrid fillers on mechanical, electrical, thermal, and other properties of silicone composites; (d) current challenges and future prospects of CNT/G/PDMS composites.

## 2. Architecture and Fabrication of CNT-Graphene Hybrid Fillers

**Figure 1:**
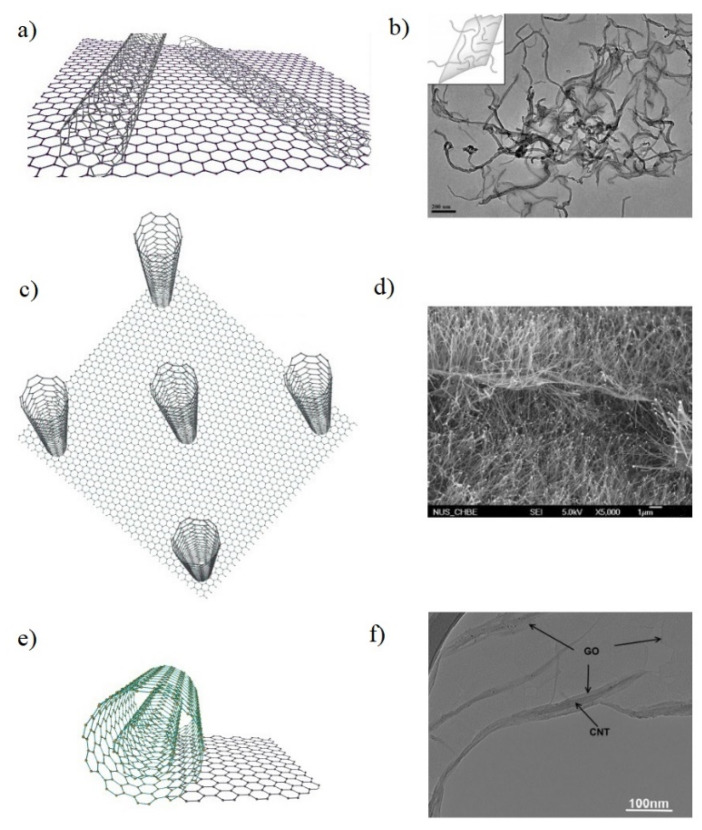
Architecture types of CNT/G hybrids: (**a**) schematic view of *h*CNT/G hybrid; (**b**) SEM picture of *h*CNT/G hybrid. Adapted from ref. [16] with permission from American Chemical Society; (**c**) schematic view of *v*CNT/G hybrid; (**d**) SEM picture of *v*CNT/G hybrid. Adapted from ref. [17] with permission from American Chemical Society; (**e**) schematic view of *w*CNT/G hybrid; (**f**) SEM picture of *w*CNT/G hybrid. Adapted from ref. [18] with permission from Elsevier Ltd.

Properties of CNT/G hybrid silicone composites depend on various factors; however, a key element of their high performance is nanoscale architecture of CNT/G hybrid fillers. Depending on the architecture type, CNT/G hybrids can be classified into three main groups (Figure 1): hybrids of graphene with horizontal CNTs (*h*CNT/G), hybrids of graphene with vertical CNTs (*v*CNT/G), and CNTs wrapped with graphene (*w*CNT/G) [15,16,17,18,19]. To obtain a hybrid structure of a certain type, two fundamentally different approaches can be used: assembly and in situ synthesis.

The assembly approach can be realized through various methods, including solution processing, layer-by-layer deposition, vacuum filtration, electrophoretic deposition, sol-gel synthesis, etc., [2,15,20]. Due to the simplicity and scalability of these methods, they are widely used to assemble *h*CNT/G and *w*CNT/G hybrids via non-covalent interactions (van der Waals, π–π stacking, electrostatic, etc.), or covalent interactions involving complementary functional groups introduced in the sp2 lattice of CNTs and graphene. However, despite the widespread use of assembly methods for CNT/G hybrid manufacturing, they have some considerable limitations including multi-step processing and poor controllability in terms of morphology, density, and orientation of hybrid structures.

To address the limitations of assembly-based methods, in situ methods (chemical vapor deposition (CVD), templated CVD, chemical unzipping, etc.) have been effectively implemented in the past decade [21,22,23]. These methods involve less processing steps, guarantee a uniform distribution of carbon allotropes, and provide an opportunity to control the nanoscale architecture of hybrid materials by adjusting the fabrication conditions. In situ methods enable the production of highly ordered *v*CNT/G, *h*CNT/G, and *w*CNT/G hybrid structures (so-called seamless hybrid structures [21]), where covalently bonded graphene and CNTs form a single, total-carbon framework with seamless C–C junctions [24,25]. Meanwhile, even though in situ methods are very effective for the fabrication of uniform hybrid materials, the required use of high processing temperatures, explosives, or toxic chemicals (B_2_H_6_, Ni (CO)_4_, etc. [26]) limits their practical application at industrial scale, so further improvements are still required.

Overall, the assembly and in situ approaches, with their pros and cons, have been successfully used to obtain CNT/G hybrid fillers designed for manufacturing of hybrid silicone composites, which are comprehensively discussed in the next section.

## 3. Fabrication and Properties of CNT/G/PDMS Composites

**Figure 2:**
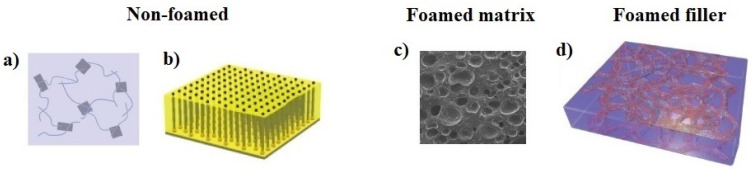
Types of CNT/G hybrid silicone composites: (**a**) composites with non-foamed matrix and unaligned fillers; (**b**) composites with non-foamed matrix and aligned fillers; (**c**) composites with foamed matrix; (**d**) composites with foamed fillers and non-foamed matrix. Adapted from ref. [27] (**a**), [28] (**b**), [29] (**c**), and [30] (**d**) with permission from Elsevier Ltd. and WILEY-VCH Verlag GmbH & Co.

Depending on a macro-scale structure of CNT/G hybrid silicone composites, they can be divided into three groups: (1) non-foamed composites, (2) composites with a foamed matrix, and (3) composites with foamed fillers (Figure 2). In the following subsections, we presented fabrication strategies for manufacturing hybrid composites of these three groups, subdividing them according to the type of hybridization (assembly or seamless junctions). For comparison purposes, we also summarized data on CNT/G/PDMS composites of different types and their fabrication techniques in Table A1 (Appendix A).

### 3.1. Non-Foamed CNT/G Hybrid Silicone Composites with Assembled Structure

**Figure 3:**
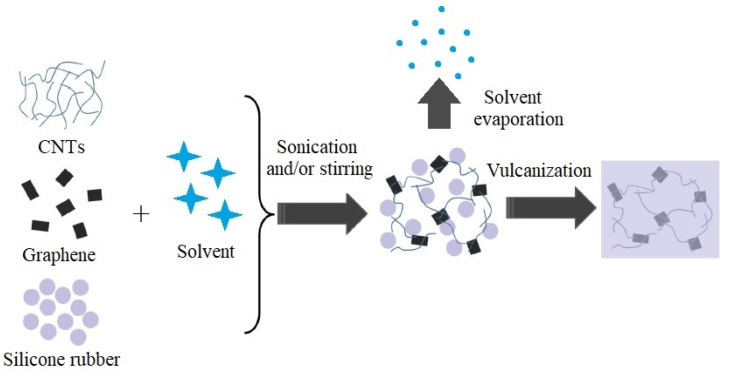
Schematic representation of the most popular approach for manufacturing of non-foamed CNT/G/PDMS composites with an assembled structure. Adapted from [27] with permission from Elsevier Ltd.

In general, non-foamed silicone composites with assembled CNT/G hybrids can be produced by solution blending in various solvents [31] or/and mechanical blending methods (ultrasonication, calendering, stirring, extrusion, high shear mixing, etc.) [32,33]. So far, the combination of solution and mechanical blending approaches is the most popular strategy for the fabrication of CNT/G/PDMS composites (Figure 3).

In particular, numerous research teams applied sonication, stirring, and solution blending in various solvents (tetrahydrofuran (THF) (Hu et al. [34], Pradhan et al. [35], Oh et al. [36]), hexane (Yang et al. [37], Kantarak et al. [38]), isopropyl alcohol (IPA) + Stoddard solvent (Lee et al. [39]), acetate (Yan et al. [40]), and toluene (Shafiei et al. [41]) to produce hybrid silicone composites with a variety of filler concentrations and CNT/G mass ratios (see Table A1). The as-prepared composites possessed the π–π stacked structure of *h*CNT/G type and demonstrated the synergistic improvement of their electrical, mechanical, and thermal properties. For instance, according to Pradhan et al. [35] and Yan et al. [40], the synergistic improvement of tensile strength, elongation at break, electrical conductivity, and electric heating performance in CNT/G/PDMS composites could reach 65, 100, 236, and 26%, respectively (see Supplementary Materials).

Another research team (Yang et al. [27]) proposed a study similar to the above studies but modified the strategy for manufacturing of CNT/G/PDMS composites. They used a combination of solution blending (in THF) and sonication processes assisted by the mixture of cetyltrimethylammonium bromide (CTAB), p-octyl polyethylene glycol phenyl ether (OP-10) compound dispersant, and sodium dodecyl sulfate (SDS) anionic surfactant. The use of CTAB, OP-10 and SDS enabled to produce hybrid composites of *h*CNT/G type with electrostatically bonded graphene and CNTs. The study reported that due to the electrostatic self-assembly, the obtained CNT/G/PDMS composites possessed a superior homogeneity and decreased percolation threshold (0.92 wt%) as compared to composites with π–π stacked CNT/G hybrid fillers (2 wt% [36] and 5 wt% [38]).

Kumpika et al. [42] also applied the modified strategy to prepare silicone composites with assembled *h*CNT/G fillers. Their two-step fabrication route involved: (1) production of CNT/G hybrid thin films through ethanol solution blending, sonication, and stirring; (2) infiltration of dried CNT/G thin films with silicone. The main advantage of this strategy over the previous ones [34,35,36,37,38,39,40,41] is the elimination of hazardous solvents that can deteriorate mechanical properties of CNT/G/PDMS composites and increase their toxicity. So the proposed technique is highly promising for the fabrication of biocompatible and durable silicone materials reinforced with graphene and CNTs.

Another eco-friendly and completely solvent-free technique was proposed by Kim et al. [43]. They used a planetary mixer to fabricate *h*CNT/G hybrid silicone composites by mechanical blending. The study of electrical and mechanical performance of as-prepared composites demonstrated their high stretchability (>100%) and prominent electrical conductivity (1 S/m) which is comparable with that of most conductive CNT/G/PDMS composites obtained through solution blending [36,40].

Our research team (Barshutina et al. [44]) also developed non-toxic, cost-effective, easy handling, and scalable technique to produce hybrid silicone composites based on few-layer graphene and MWCNTs. The proposed technique involves the fabrication of CNT/G hybrid fillers by water solution blending and manufacturing of CNT/G/PDMS composites through calendering in a three-roll mill. This strategy enables to obtain hybrid composites with a prominent electrical conductivity (up to 1 S/m), high stretchability (up to 100%), good durability (>1000 strain cycles), and prominent electrical stability under a cyclic loading at 30% strain. Besides, our studies demonstrated a good biocompatibility of as-prepared CNT/G/PDMS composites, which is highly promising for biosensors and bioelectronics applications.

Overall, due to their unique properties, silicone composites with assembled CNT/G hybrid fillers are considered as promising materials to produce strain sensors for health monitoring [27,37,38,39,42,43,44], flexible electric heating elements [40], selective membranes [41], electromagnetic interference (EMI) shielding coatings [34,35], etc.

### 3.2. Non-Foamed CNT/G Hybrid Silicone Composites with Seamless Structure

The seamless CNT/G hybrids obtained typically by CVD or heat treatment techniques have a highly ordered or even aligned structure that can be significantly disrupted by conventional mechanical and solution blending methods. For this reason, the fabrication of non-foamed silicone composites with seamlessly bonded CNTs and graphene is mainly performed by various infiltration methods (dip-coating, drop casting, vacuum impregnation, etc.), which enable the infusion of silicone into CNT/G hybrid structures without affecting their order and alignment.

**Figure 4:**
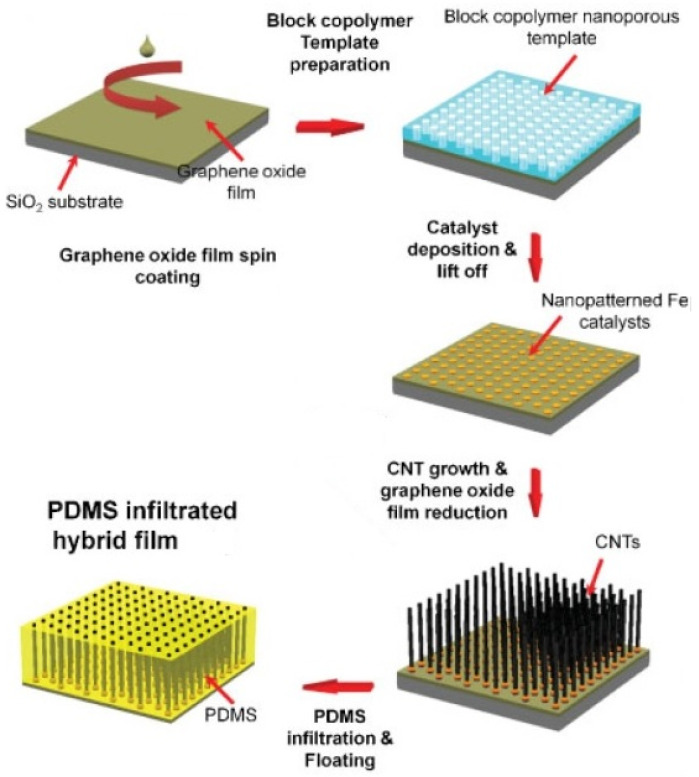
Schematic representation of the proposed by Lee et al. [28] technique for manufacturing non-foamed CNT/G/PDMS composites with a seamless structure. Adapted from [28] with permission from WILEY-VCH Verlag GmbH & Co.

For instance, Lee et al. [28] used a combination of in situ CVD and infiltration processes to prepare thin films of silicone composites based on seamlessly bonded graphene and few-walled CNTs. In the fabrication route (Figure 4), vertically aligned CNTs were grown from the iron catalyst on GO surfaces via plasma-enhanced CVD process, which also promoted the thermal reduction of graphene oxide to a highly conductive graphene. Subsequently, the as-prepared CNT/graphene hybrid structures were infiltrated with silicone by drop casting, while the infiltration thickness was precisely controlled to leave the top ends of CNTs exposed. The obtained composites exhibited prominent mechanical and field-emission properties. In particular, the values of field-enhancement factor (14,500) and turn-on voltage (0.4 V/μm) are among the best results reported for carbon-based field emitters [45].

A similar but reverse strategy was proposed by Shi et al. [46]. They first synthesized ultrathin CNTs films by floating CVD, and then used them as porous templates for CVD growth of graphene. The obtained hybrid fillers with a seamless structure of *h*CNT/G type were further infiltrated with silicone to produce CNT/G/PDMS composite films. The authors demonstrated the effectiveness of their approach for manufacturing strain sensing materials with linear and reliable resistance response to strain, which is due to the strong interaction and effective load transfer within CNT/G hybrid silicone films.

An alternative fabrication approach was used by Zhao et al. [47] to produce CNT/G/PDMS composites with seamlessly bonded graphene and CNTs. At the first stage, they applied solution blending and ultrasonication methods for manufacturing of assembled *h*CNT/G hybrid fillers, which were then converted into seamless ones by annealing at 1050 °C. Subsequently, CNT/G hybrid silicone composites were prepared by ethyl acetate solution blending and magnetic stirring. The proposed strategy enabled to obtain seamlessly hybridized and thermally conductive silicone composites without using complicated CVD process; however, it provides poor fabrication control in terms of morphology, density, and orientation of hybrid structures.

Overall, according to the above studies, non-foamed CNT/G/PDMS composites with a seamless structure are highly potential materials for various high-technological applications including field-emission, strain sensing, and thermal electronics. However, the number of scientific papers in this area is still limited, so further comprehensive research is highly required.

### 3.3. Silicone Composites with a Foamed Matrix and Assembled CNT/G Hybrid Fillers

According to reviewed literature, all CNT/G hybrid composites with a foamed silicone matrix are based on assembled CNT/G hybrid fillers, which is probably due to the complexity of combining in situ and silicone foaming processes in the one strategy. In general, silicone foaming can be performed (1) by using a foaming agent, (2) through a chemical reaction leading to gas evolution, or (3) by means of template techniques.

**Figure 5:**
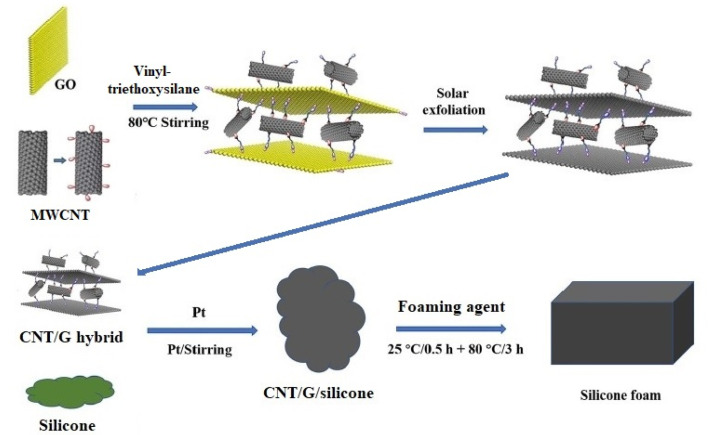
Schematic illustration of the proposed by Zhang et al. [29] approach for the fabrication of CNT/G/PDMS with a foamed matrix and assembled hybrid fillers. Adapted from ref. [29] with permission from WILEY-VCH Verlag GmbH & Co.

The first approach was used by Zhang et al. [29] to prepare CNT/G hybrid silicone composites with a foamed matrix. At the first stage, they produced functionalized CNT/G hybrids via aqueous solution blending in the presence of vinyltriethoxysilane, and then foamed CNT/G/PDMS composites were fabricated by mechanical blending of obtained hybrid fillers with silicone rubber and a foaming agent (polyhydroxysiloxane) (Figure 5). The authors demonstrated that the joint use of a foaming agent and CNT/G hybrid fillers functionalized with vinyl groups promoted the formation of highly homogeneous foamed silicone composites with improved tensile strength (by 70%) and thermal conductivity (by 204%) as compared to pure silicone foam.

The second approach based on a chemical reaction leading to gas evolution was used by Valentini et al. [48]. They prepared foamed CNT/G/PDMS composites by using the simultaneous reactions of beer yeast fermentation and silicone gelation. To initiate the fermentation process, an aqueous solution of beer yeast and sugar was added to a magnetically stirred mixture of multi-walled CNTs, graphene, and PDMS. As a result, the porous composite structure was formed by CO_2_ bubbles accumulated in the gelling silicone matrix. The proposed strategy enabled to obtain multifunctional CNT/G/PDMS composites with unique biological properties and auxetic behavior, which are the key characteristics for several specific applications (fasteners, plast damping, medical implants, etc., [49]).

Another research team (Chen et al. [50]) applied a template-based approach to produce *h*CNT/G hybrid silicone composites with a foamed matrix. Their fabrication strategy involved: (a) manufacturing of CNT/G hybrid fillers via aqueous solution blending and ultrasonication; (b) forming a 3D porous silicone scaffold by replicating the structure of a sacrificial nickel foam template; (c) assembly of CNT/G hybrid fillers on the silicone scaffold using the solution impregnation technique. The as-prepared composites exhibited the synergistically improved electrical conductivity by up to 284% (see Supplementary Material), low percolation threshold (0.2 wt%), and superior electro-mechanical stability under cyclic bending and stretching. In order to clarify the superior properties of foamed CNT/G/PDMS composites, the authors also developed and verified a theoretical model simulating the deformation modes of a porous composite structure under tensile loads.

The same fabrication route was used by Duan et al. [51] for manufacturing foamed silicone composites with *h*CNT/G hybrid fillers. However, instead of nickel foam templates, the authors used 3D printed split-level and aligned scaffolds made of polylactic acid (PLA) that is known for its biocompatibility and ease of removal with organic solvents. The study demonstrated the superior efficiency of split-level PLA templates over the aligned ones to produce CNT/G/PDMS composites with stable electrical performance under cyclic bending and stretching deformations. Besides, due to the unique porous structure, these composites exhibited an excellent stretchability of up to 340%, which is the best result reported so far for CNT/G hybrid silicone composites.

Summing up the above studies, we can conclude that CNT/G/PDMS composites with a foamed matrix can exhibit prominent electrical conductivity and versatile mechanical properties (e.g., lightness, flexibility, elasticity, negative Poisson’s ratio, and damage resistance), which are highly promising for next-generation stretchable electronics, such as E-skins, bioimplants, wearable electronics, etc.

### 3.4. Silicone Composites with Foamed and Seamlessly Bonded CNT/G Hybrid Fillers

The foamed CNT-graphene hybrid fillers can be obtained through various methods including hydro/solvothermal reduction, sol-gel synthesis, microfluidic technique, direct freeze-drying, template-directed CVD synthesis, template directed electron deposition, etc., [52,53]. The application of these methods enables the formation of highly ordered and 3D interconnected porous structures with covalently or non-covalently bonded CNTs and graphene. When used as fillers for silicone composites, these porous structures function as a supporting skeleton that is filled with liquid silicone by one of the infiltration methods (dip-coating, spin-coating, bar-coating, drop casting, vacuum impregnation, etc., [54,55]).

**Figure 6:**
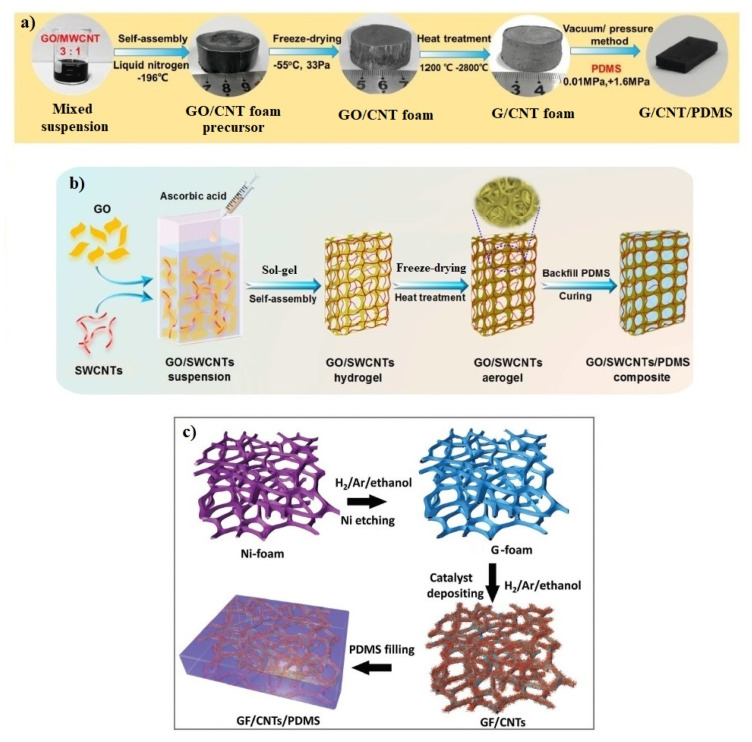
Manufacturing scheme to produce CNT/G/PDMS composites with foamed and seamlessly bonded fillers. (**a**) Adapted from ref. [29] with permission from Elsevier Ltd.; (**b**) Adapted from ref. [56] with permission from American Chemical Society; (**c**) Adapted from ref. [57] with permission from WILEY-VCH Verlag GmbH & Co.

For instance, Jia et al. [30] applied a combination of ethanol solution blending, freeze-drying, and annealing (at >1200 °C) to prepare *h*CNT/G hybrid scaffolds with a seamless structure, which were subsequently used for manufacturing of hybrid silicone composites by a vacuum-infiltration method (Figure 6a). The authors noted that the non-covalent bonds between graphene and multi-walled CNTs were converted to the seamless covalent bonds after the annealing process, which significantly contributed to the synergistic improvement of hybrid composite performance. In particular, the obtained composites demonstrated a prominent electrical conductivity (>100 S/m) and high specific EMI shielding effectiveness (87.86 dB·cm^3^/g), which was improved by 141% compared to that of G/PDMS composites.

A similar but modified strategy was used by Zhao et al. [56] to produce silicone composites with foamed hybrid fillers based on graphene and single-walled CNTs. Their fabrication route involved sol-gel self-assembly (assisted by L-ascorbic acid), freeze-drying, annealing (at 800 °C), and vacuum-infiltration processes (Figure 6b). The study reported that as-prepared CNT/G/PDMS composites even at ultra-low filler loading (0.25–0.35 wt%) outperformed single-filler composites prepared by conventional blending methods with high concentrations (10–15 wt%) of carbon fillers. In particular, the most significant improvement (by several orders of magnitude) was achieved for electrical conductivity (120 S/m) and specific EMI shielding effectiveness (110 dB·cm^3^/g) over the X-band frequency range.

Chen et al. [58] applied almost the same strategy based on sol-gel synthesis (assisted by resorcinol and formaldehyde precursors), freeze-drying, pyrolysis (at 1000 °C), and vacuum-infiltration techniques. The study reported that electrical conductivity (280 S/m) of as-prepared composites was improved by more than four orders of magnitude compared to that of identical composites prepared by solution blending. Besides, the electrical conductivity retention rate of CNT/G/PDMS films was nearly 2.5 and 6.8 times higher than that of CNT/PDMS and G/PDMS films at 30% strain.

A different approach was used by Kong et al. [59] for the manufacturing of hybrid silicone composites with foamed fillers of *v*CNT/G type. They used water solution blending, freeze-drying, and heat-reduction processes to prepare a foamed graphene skeleton, which was subsequently used as a template for the in situ growth of multi-walled CNTs by a low-temperature CVD. Further, the as-prepared CNT/G hybrids were purified and incorporated into silicone by a simple mechanical stirring method. The authors reported that their fabrication route is highly effective to produce light weight and high-performance EMI shielding composites with a prominent electromagnetic reflection coefficient (−55 dB) and wide absorption bandwidth (3.5 GHz).

Another effective strategy was applied by Cai et al. [57] to prepare silicone composites with foamed *v*CNT/G hybrid fillers. They used the Ni template-directed CVD method for the manufacturing of graphene foams, which were then seamlessly hybridized with CNTs by another CVD process (Figure 6c). Subsequently, CNT/G hybrid foams were infiltrated with silicone and used to fabricate flexible and reversible strain sensors. The study investigated the performance of as-prepared hybrid sensors under different deformations (stretching, bending, torsion, etc.) and revealed its remarkable improvement (up to 70%) compared to G/PDMS sensors.

Overall, according to the reviewed studies, silicone composites with foamed and seamless CNT/G hybrid fillers demonstrate prominent electro-mechanical and electromagnetic properties, which are highly beneficial for wearable electronics [57,58] and EMI shielding applications [30,56,59].

### 3.5. Silicone Composites with Foamed and Assembled CNT/G Hybrid Fillers

There are only a few studies on CNT/G/PDMS composites with foamed and assembled hybrid fillers; however, they reported highly outstanding results. For instance, Sun et al. [60] proposed an effective strategy to produce hybrid silicone composites with remarkable electrical and electromagnetic properties. Their fabrication route involved (a) the preparation of graphene foam via Ni template-directed CVD, (b) producing a mixture of ethyl acetate-diluted PDMS and CNTs by solution blending and ultrasonication, (c) manufacturing of hybrid silicone composites through infiltration of CNT/PDMS mixture into graphene foam. The study reported that electrical conductivity of as-prepared composites was synergistically improved by 322% (see Supplementary Material) and attained the value of 3150 S/m, which is so far the best result for CNT/G/PDMS composites. Besides, the specific EMI shielding effectiveness of these composites reached 833 dB·cm^3^/g, which is among the highest for carbon-based EMI shielding composites [61].

**Figure 7:**
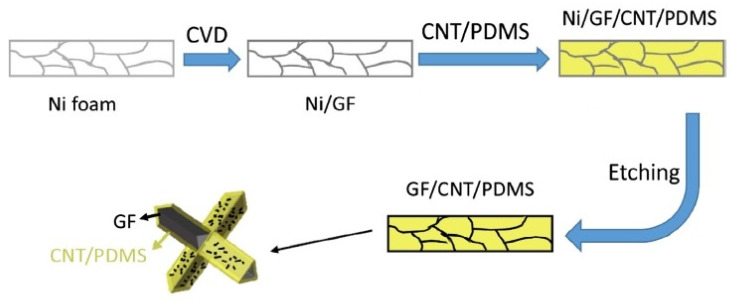
Fabrication scheme to produce CNT/G/PDMS composites with foamed and assembled hybrid fillers. Adapted from ref. [62] with permission from Elsevier Ltd.

A similar fabrication strategy was used by (Wu et al. [62]) to fabricate sound absorbing materials based on CNT/G/PDMS composites (Figure 7). The study demonstrated that a sound absorption coefficient of these materials can reach 0.3 in an expanded frequency range from 100 to 1000 Hz, which is promising for low-frequency noise shielding applications.

## 4. Summary and Perspectives

The analysis of numerous studies on CNT/G hybrid silicone composites indicated that significant progress has been made in this field over the past 10 years. The existing fabrication techniques enable to obtain various configurations of CNT/G/PDMS composites distinguished by (a) the nanoscale architecture of hybrid filler (*v*CNT/G, *h*CNT/G, or *w*CNT/G), (b) type of hybridization (assembled or seamless hybrids); (c) macroscale structure of filler (foamed or non-foamed); (d) and macroscale structure of matrix (foamed or non-foamed). According to the reviewed literature, CNT/G/PDMS composites of different configurations exhibited prominent electrical, mechanical, electromagnetic, and thermal properties, which are promising for multiple applications.

However, the widespread use of these materials is hindered by following major challenges. First one is the need to conduct thorough research on hybridization and synergistic mechanisms in CNT/G hybrid materials obtained by different techniques. A better understanding of the nature of these mechanisms is crucial for the development of computational models that can predict the structural parameters and functional properties of the hybrid composites, depending on the fabrication strategy and processing conditions.

A second major challenge is the development of effective, scalable, and safe techniques for the fabrication of seamless and highly ordered CNT/G hybrid fillers, which have a more pronounced synergistic effect on silicone composite properties than assembled hybrid fillers. Currently, seamless CNT/G hybrids are produced by methods that require the use of high processing temperatures, explosive gases, and toxic chemicals, so a shift toward environmentally friendly and safe technologies is highly needed. Besides, the elimination of toxic and hazardous chemicals in the fabrication process is essential for hybrid materials of biomedical applications.

Another important challenge is increasing the electrical conductivity of CNT/G/PDMS composites to a level required for advanced microelectronics. Even though the conductivity of hybrid silicone composites is synergistically improved as compared to single-filler composites, its values are still not sufficient enough. To overcome this challenge, the following approaches could be used: (a) doping of CNT/G hybrid fillers with heteroatoms such as nitrogen to improve their intrinsic conductivity; (b) the development of new techniques that enable to introduce high concentrations (>10 wt%) of CNT/G hybrid fillers in silicone matrices without the agglomeration effect; (c) the improvement of existing techniques for the fabrication of CNT/G/PDMS composites with an aligned and 3D interconnected conductive network structure.

The last but not least challenge is decreasing the production cost of CNT/G hybrid fillers and their polymer composites. The non-availability of high quality CNTs and graphene in large volumes and at low prices is a key factor preventing the widespread use of their composites in industry, medicine, and daily life. To address this issue, the development of cost-effective, scalable, and facile manufacturing methods is required.

Overall, we hope that our review will help to build a complete picture of existing approaches for the manufacturing of CNT/G hybrid silicone composites, take a deeper look at current challenges and achievements, and inspire some scientists to make breakthrough research in this field.

## Data Availability

The data presented in this study are available on request from the corresponding author.

## Supplementary Materials

The following are available online at https://www.mdpi.com/article/10.3390/ma14092418/s1.

## Appendix A

**Table A1:** Summary on CNT/G/PDMS composites.

Filler *	Matrix	CNT/G Ratio *	Filler Loads *, (wt%)	Fabrication Techniques	Key Properties	Applications	Ref.
non-foamed assembled *h*MWCNT/G	non- foamed PDMS	2:1 **2.5:1** **3:1**	3.0 **3.5** **4.0**	THF solution blending/ sonication	σ*_e_* = 1.25 mS/m Δ = 0.123 m^2^/s	electrostatic discharge, EMI shielding.	[34]
		1:1	0.375 0.5 **0.75** **1.0** 1.5	THF solution blending/ sonication	σ*_ts_* = 0.67 MPa ε = 194% *T*_0_ = 441 °C *q*_800_ = 56%	EMI shielding, flame retarding	[35]
		1:1 7:3 **9:1**	0.4 **0.6** 1.0	THF solution blending/ sonication	σ*_e_* = 0.617 S/m ε = 215% ε*_r_* = 60%	stretchable electronics	[36]
		3:1	0.5 1.0 1.5 2.0 **3.0** 4.0	THF solution blending/ sonication	*p_c_* = 0.92 wt% GF = 19.2 ε*_r_* = 30%	strain sensing for health monitoring	[27]
		2:1	1.0 2.0 3.0 **4.0** 5.0	*n*-hexane solution blending/sonication/ stirring	*p_c_* = 2.0 wt% GF = 11.6 ε*_r_* = 30%	strain sensing for health monitoring	[37]
		1:1	9.0	hexane solution blending/stirring	GF = ~4.4 ε = ~70% ε*_r_* = 33%	strain sensing for tremor detection	[38]
		3.5:1.5	5.0	IPA + Stoddard solvent solution blending/ sonication	*p_c_* = 5.0 wt% GF = > 100 ε*_r_* = 40%	strain sensing for health monitoring	[39]
		1:9 3:7 1:1 7:3 **9:1**	1.0	acetate solution blending/ultrasonication	σ*_e_* = 1.37 S/m *h_rc_* = 7.46 mW/°C *T*_10_ = 488 °C *q*_800_ = 56%	flexible electric heating elements	[40]
		1:3	0.25 0.5 **0.75** 1.0	toluene solution blending/ultrasonication/stirring	*k* = 22.5	selective membranes for ethanol-water separation	[41]
		1:3 **1:1** 3:1	n/a	ethanol solution blending/sonication/ stirring/infiltration	GF = 10.9 ε = 71% ε*_r_* = 33%	strain sensing for gait monitoring	[42]
		6:4 7:3 8:2 **9:1**	1.0	planetary mixing	σ*_e_* = 1 S/m ε = ~100% ε*_r_* = 30%	conductive dry adhesives for ECG monitoring	[43]
		1:9 **2:8** 4:6 6:4 8:2	10.0 **15.0** 20.0	aqueous solution blending/stirring/ ultrasonication/ calendering in a three-roll mill	σ*_e_* = 0.6 … 1 S/m ε = 60 … 100% ε*_r_* = 30%	biosensors and bioelectronics	[44]
non-foamed seamless *v*(**2**, 3, or 6) WCNT/G		n/a	n/a	plasma-enhanced CVD/ infiltration	β = 14500 *E_to_* = 0.4 V/ μ m ε*_r_* = 45%	field-emission stretchable electronics	[28]
non-foamed seamless *h*CNT/G		n/a	n/a	CVD/ infiltration	GF = ~0.36 ε*_r_* = 20%	strain sensing for wearable electronics	[46]
non-foamed seamless *h*MWCNT/G		1:4	0.25 0.5 0.75 **1.0** 2.0 **3.0**	aqueous solution blending/ultrasonication/annealing/acetate solution blending/ magnetic stirring	σ*_e_* = 2 mS/m λ = 0.29 W/m·K *T_0_* = 419 °C	conductive and thermal management elastomer materials	[47]
non-foamed assembled *h*MWCNT/G	foamed PDMS	1:4 **1:1** 4:1	~2.0	aqueous solution blending/stirring/ foaming	σ*_e_* = 0.12 mS/m λ = 0.548 W/m·K σ*_ts_* = 0.6 MPa ε = 110%	stretchable and soft electronics	[29]
		1:3 **1:1** 3:1	1.0	aqueous solution blending/stirring/ fermentation	σ*_e_* = 1.4 nS/m ε = 96% σ*_ts_* = 0.17 MPa	biomedical stretchable electronics	[48]
		1:1	2.0	aqueous solution blending/ultrasonication/Ni template replication/ forced infiltration	σ*_e_* = 27 S/m *p_c_* = 0.2 wt% ε*_r_* = 50%	next-generation stretchable electronics	[50]
		1:1	1.0	aqueous solution blending/ultrasonication/PLA template replication/infiltration	σ*_e_* = 5.12 S/m ε = 340% ε*_r_* = 50%	next-generation stretchable electronics	[51]
foamed seamless *h*SWCNT/G	non- foamed PDMS	1:6 1:5 **1:3** 1:1	0.25 0.27 **0.28** 0.35	sol-gel self-assembly/ ultrasonication/stirring/ freeze-drying/annealing/ vacuum backfilling	σ*_e_* = 120 S/m SE = 31 dB SSE = 110 dB·cm^3^/g *R_S_* = 7.94 MPa	EMI shielding	[56]
foamed seamless *h*MWCNT/G		1:3	0.95 0.96 0.97 **0.98** 1.03 1.05	ethanol solution blending/ultrasonication/freeze-drying/annealing/ vacuum infiltration	σ*_e_* = 100.99 S/m SE = 54.43 dB SSE = 87.86 dB·cm^3^/g *R_S_* = 3.34 MPa λ = 0.29 W/m·K	EMI shielding	[30]
		1:1	1.3	sol-gel synthesis/ ultrasonication/freeze-drying/pyrolysis/ vacuum infiltration	σ*_e_* = 280 S/m ε*_r_* = 20%	electronic textiles and smart clothing	[58]
foamed seamless *v*MWCNT/G		n/a	2.5 **5.0** **10.0**	aqueous solution blending/ freeze-drying/CVD/stirring	Γ = −55 dB SE = 10 dB	EMI shielding	[59]
		n/a	n/a	Ni template-directed CVD/CVD/infiltration	GF = 35 ε*_r_* = 85%	strain sensing for wearable electronics, health monitoring, etc.	[57]
foamed assembled *h*MWCNT/G		2:2.7	4.7	Ni template-directed CVD/ethyl acetate solution blending/ ultrasonication/ infiltration	σ*_e_* = 3150 S/m SE = 75 dB SSE = 833 dB·cm^3^/g	EMI shielding	[60]
		1:1	2.0	Ni template-directed CVD/ethyl acetate solution blending/ sonication/ stirring/infiltration	α = 0.3	low-frequency noise shielding	[62]

*—optimal values are highlighted in bold; σ*_e_*—electrical conductivity; *p_c_*—percolation threshold; Δ—thermal diffusivity; λ—thermal conductivity; ε—elongation at break; ε*_r_*—reliable strain; σ*_ts_*—tensile strength; *R_S_*—compressive strength; GF—gauge factor; *h_rc_*—heat transferred by radiation and convection (electric heating performance); *T*_0_—initial degradation temperature; *T*_10_—temperature corresponding to 10% weight loss; *q*_800_—char residue at 800 °C; *k*—separation factor; β—field-enhancement factor; *E_to_*—turn-on voltage; Γ—electromagnetic reflection coefficient; *SE*—electromagnetic interference shielding effectiveness; SSE—specific EMI shielding effectiveness; α—sound absorption coefficient.
