# Supplementary material for: Silicone Composites with CNT/Graphene Hybrid Fillers: A Review
Source: Materials (Basel). 2021 May 6;14(9):2418. doi: 10.3390/ma14092418 (PMC8124395; doi:10.3390/ma14092418)
Supplement: Supplementary file 1 [file materials-14-02418-s001.zip › materials-1176332-supplementary.pdf]

materials

Supplementary

Silicone Composites with CNT/Graphene Hybrid Fillers: A Review

Marie N. Barshutina [1,4,*], Valentyn S. Volkov [1,*], Aleksey V. Arsenin [1], Albert G. Nasibulin [2,3], Sergey N. Barshutin [5], Alexey G. Tkachev [4,5]

1 Center for Photonics and 2D Materials, Moscow Institute of Physics and Technology, Dolgoprudny 141700, Russia; arsenin.av@mipt.ru
2 Skolkovo Institute of Science and Technology, Nobel Str. 3, Moscow 121205, Russia; a.nasibulin@skoltech.ru
3 School of Chemical Engineering, Aalto University, P.O. Box 16100, Aalto 00076, Finland
4 NanoTechCenter Ltd., Tambov 392000, Russia; postmaster@kma.tstu.ru
5 Tambov State Technical University, Tambov 392000, Russia; barshutin.sn@tstu.ru
* Correspondence: barshutina.mn@mipt.ru (M.N.B.), volkov.vs@mipt.ru (V.S.V.)

To assess the synergistic improvement in the parameters of CNT/G/PDMS composites, their values were compared with the theoretical ones (α(t)) calculated from the additive model:

$$a(t) = \varphi_{CNT} \times a(CNT/PDMS) + \varphi_G \times a(G/PDMS) \quad (1)$$

wherein φ_{CNT} is the fraction of CNTs in hybrid filler; φ_G is the fraction of graphene in hybrid filler; $\alpha_{CNT/PDMS}$ is an experimental value of the target parameter for CNT/PDMS composites at *X*% filler loading; $\alpha_{G/PDMS}$ is an experimental value of the target parameter for G/PDMS composites at *X*% filler loading.

The total relative improvement was then found as

$$\delta = \frac{a(exp) - a(t)}{a(t)} \times 100\% \quad (2)$$

wherein α(exp) is an experimental value of the target parameter for CNT/G/PDMS composites at *X*% filler loading.

In this way, we calculated the synergistic improvement of tensile strength (σ_{ts}), elongation at break (ε), electrical conductivity (σ_e), electric heating performance (h_{rc}), thermal conductivity (λ) using data from

Pradhan et al. [1]:

$$\delta(\sigma_{ts}) = \frac{\sigma_{ts}(exp) - (\varphi_{CNT} \times \sigma_{ts}(CNT/PDMS) + \varphi_G \times \sigma_{ts}(G/PDMS))}{\varphi_{CNT} \times \sigma_{ts}(CNT/PDMS) + \varphi_G \times \sigma_{ts}(G/PDMS)} \times 100\% \quad (3)$$

$$\delta(\sigma_{ts}) = \frac{0.67 - (0.5 \times 0.44 + 0.5 \times 0.37)}{0.5 \times 0.44 + 0.5 \times 0.37} \times 100\% \approx 65\% \quad (4)$$

$$\delta(\varepsilon) = \frac{\varepsilon(exp) - (\varphi_{CNT} \times \varepsilon(CNT/PDMS) + \varphi_G \times \varepsilon(G/PDMS))}{\varphi_{CNT} \times \varepsilon(CNT/PDMS) + \varphi_G \times \varepsilon(G/PDMS)} \times 100\% \quad (5)$$

$$\delta(\varepsilon)=\frac{194-(0.5\times 104+0.5\times 90)}{0.5\times 104+0.5\times 90}\times 100\%=100\% \tag{6}$$

Yan et al. [2]:

$$\delta(\sigma_e)=\frac{\sigma_e(exp)-\ (\varphi_{CNT}\times\sigma_e(CNT/PDMS)+\varphi_G\times\sigma_e(G/PDMS))}{\varphi_{CNT}\times\sigma_e(CNT/PDMS)+\varphi_G\times\sigma_e(G/PDMS)}\times 100\% \tag{7}$$

$$\delta(\sigma_e)=\frac{1.37-(0.9\times 0.435+0.1\times 0.161)}{0.9\times 0.435+0.1\times 0.161}\times 100\%\approx 236\% \tag{8}$$

$$\delta(\mathrm{h_{rc}})=\frac{\mathrm{h_{rc}(exp)}-\ (\varphi_{\mathrm{CNT}}\times\mathrm{h_{rc}(CNT/PDMS)}+\varphi_{\mathrm{G}}\times\mathrm{h_{rc}(G/PDMS)})}{\varphi_{\mathrm{CNT}}\times\mathrm{h_{rc}(CNT/PDMS)}+\varphi_{\mathrm{G}}\times\mathrm{h_{rc}(G/PDMS)}}\times 100\% \tag{9}$$

$$\delta(h_{rc})=\frac{7.46-(0.9\times 5.79+0.1\times 6.89)}{0.9\times 5.79+0.1\times 6.89}\times 100\%\approx 26\% \tag{10}$$

Chen et al. [3]:

$$\delta(\sigma_e)=\frac{\sigma_e(exp)-\ (\varphi_{CNT}\times\sigma_e(CNT/PDMS)+\varphi_G\times\sigma_e(G/PDMS))}{\varphi_{CNT}\times\sigma_e(CNT/PDMS)+\varphi_G\times\sigma_e(G/PDMS)}\times 100\% \tag{11}$$

$$\delta(\sigma_e)=\frac{27-(0.5\times 0.07+0.5\times 14)}{0.5\times 0.07+0.5\times 14}\times 100\%\approx 284\% \tag{12}$$

Sun et al. [4]:

$$\delta(\sigma_\mathrm{e})=\frac{\sigma_\mathrm{e}(\exp\,(4.7\%))-\ (\sigma_\mathrm{e}(\mathrm{CNT}(2\%)/\mathrm{PDMS})+\sigma_\mathrm{e}(\mathrm{G}(2.7\%)/\mathrm{PDMS}))}{\sigma_\mathrm{e}(\mathrm{CNT}(2\%)/\mathrm{PDMS})+\sigma_\mathrm{e}(\mathrm{G}(2.7\%)/\mathrm{PDMS})}\times 100\% \tag{13}$$

$$\delta(\sigma_e)=\frac{3150-(73+674)}{73+674}\times 100\%\approx 322\% \tag{14}$$

References

1. Pradhan, B.; Srivastava, S. Synergistic effect of three-dimensional multi-walled carbon nanotube–graphene nanofiller in enhancing the mechanical and thermal properties of high-performance silicone rubber. *Polym. Int.* **2014**, *63*, 1219–1228.
2. Yan, J.; Jeong, Y.G. Synergistic effect of hybrid carbon fillers on electric heating behavior of flexible polydimethylsiloxane-based composite films. *Compos. Sci. Technol.* **2015**, *106*, 134–140.
3. Chen, M.; Zhang, L.; Duan, S.; Jing, S.; Jiang, H.; Li, C. Highly stretchable conductors integrated with a conductive carbon nanotube/graphene network and 3D porous poly(dimethylsiloxane). *Adv. Funct. Mater.* **2014**, *24*, 7548–7556.
4. Sun, X.; Liu, X.; Shen, X.; Wu, Y.; Wang, Z; Kim, J. Reprint of graphene foam/carbon nanotube/poly(dimethyl siloxane) composites for exceptional microwave shielding. *Compos. Part A Appl. Sci. Manuf.* **2017**, *92*, 190–197.
